# Integrative taxonomy and phylogeography of *Telenomus remus* (Scelionidae), with the first record of natural parasitism of *Spodoptera* spp. in Brazil

**DOI:** 10.1038/s41598-021-93510-3

**Published:** 2021-07-08

**Authors:** Ana P. G. S. Wengrat, Aloisio Coelho Junior, Jose R. P. Parra, Tamara A. Takahashi, Luis A. Foerster, Alberto S. Corrêa, Andrew Polaszek, Norman F. Johnson, Valmir A. Costa, Roberto A. Zucchi

**Affiliations:** 1 Department of Entomology and Acarology, University of São Paulo (USP)/Luiz de Queiroz College of Agriculture (ESALQ), Piracicaba, São Paulo Brazil; 2 Department of Phytotechnics and Plant Health, Federal University of Paraná, Curitiba, Paraná Brazil; 3 Departament of Zoology, Federal University of Paraná, Curitiba, Paraná Brazil; 4 Department of Life Sciences, Natural History Museum, Cromwell Road, London, SW7 5BD UK; 5 Department of Evolution, Ecology, and Organismal Biology, The Ohio State University, 1315 Kinnear Road, Columbus, OH 43212 USA; 6 Instituto Biológico, Unidade Laboratorial de Referência em Controle Biológico, Campinas, São Paulo Brazil

**Keywords:** Zoology, Animal behaviour, Entomology

## Abstract

The egg parasitoid *Telenomus remus* (Hymenoptera: Scelionidae) has been investigated for classical and applied biological control of noctuid pests, especially *Spodoptera* (Lepidoptera: Noctuidae) species. Although *T. remus* was introduced into Brazil over three decades ago for classical biological control of *S. frugiperda*, this wasp has not been recorded as established in corn or soybean crops. We used an integrative approach to identify *T. remus*, combining a taxonomic key based on the male genitalia with DNA barcoding, using a cytochrome c oxidase subunit I mitochondrial gene fragment. This is the first report of natural parasitism of *T. remus* on *S. frugiperda* and *S. cosmioides* eggs at two locations in Brazil. We also confirmed that the *T. remus* lineage in Brazil derives from a strain in Venezuela (originally from Papua New Guinea and introduced into the Americas, Africa, and Asia). The occurrence of *T. remus* parasitizing *S. frugiperda* and *S. cosmioides* eggs in field conditions, not associated with inundative releases, suggests that the species has managed to establish itself in the field in Brazil. This opens possibilities for future biological control programs, since *T. remus* shows good potential for mass rearing and egg parasitism of important agricultural pests such as *Spodoptera* species.

## Introduction

The genus *Spodoptera* (Guenée, 1852) (Lepidoptera: Noctuidae) includes polyphagous species that have high dispersal capacities and reproductive rates and are important agricultural pests worldwide^1,2^. In the Americas, three species, *S. cosmioides* (Walker, 1858), *S. eridania* (Cramer, 1782), and *S. frugiperda* (J. E. Smith, 1797), are the most harmful crop pests^3,4^. *S. frugiperda* is particularly serious because of frequent population outbreaks in corn (maize), soybean, and cotton crops^1,2,5,6^. *Spodoptera frugiperda* was first reported in Africa in 2016 and subsequently in Asia and Oceania, and has become a serious cosmopolitan pest of corn, rice and other cereal crops^7,8^.

Insecticides and Bt crops are the most frequent methods used to control *Spodoptera* species^9–11^. However, several instances of resistance to insecticides and Bt plants have been reported, mainly for *S. frugiperda*^12,13^. In recent years, biological control has gained prominence for the control of *Spodoptera*, mainly integrated with Bt crops where insecticides are less frequently applied.

Among the natural enemies of *Spodoptera,* egg parasitoids of the genus *Telenomus* Haliday, 1833 (Hymenoptera: Scelionidae) are potential biological control agents^14,15^. Accurate identification of *Telenomus* species is complicated because of their close morphological similarity. Although characters of the male genitalia are the most reliable features, identification, especially of the *californicus* group species, requires extensive revision because of the existence of cryptic and yet undescribed species, particularly in tropical regions^16^. Considering that correct identification of biological control agents underpins the success of a biological control program^17,18^, molecular identification is an alternative method to clarify species identity within the *californicus* group.

*Telenomus remus* Nixon, 1937, native to peninsular Malaysia and Papua New Guinea, was introduced into South and Central America as a classical biological control agent against *Spodoptera* spp.^19^. Recently, *T*. *remus* was reported as naturally parasitizing *Spodoptera* species in North America and Africa^16^.

The first introduction of *T. remus* into Brazil, in 1983, was a strain from the Dominican Republic intended for classical biological control of *S. frugiperda*^14^. Subsequently, the Centro de Pesquisa de Milho e Sorgo (Sete Lagoas, Minas Gerais) introduced individuals from Venezuela in 1996. A third introduction was conducted in 2011 by the Universidade Estadual Paulista, Campus of Jaboticabal, also using wasps from Venezuela^20^. These parasitoids were collected in Barquisimeto, Lara, Venezuela, in *S. frugiperda* eggs on corn. The *T. remus* strain reared and released in Venezuela originated from Papua New Guinea^14^.

Although *T. remus* was first introduced in Brazil over 35 years ago, its natural occurrences have not been reported, indicating an incomplete or complete failure to establish in the Brazilian agricultural ecosystems. However, a recent collection of a *Telenomus* species alerted us to the presence of *T. remus* in the field. Here, we describe the identification of *T. remus*, using combined taxonomic methods, and confirm, through a phylogeographic approach, the origin of the *T. remus* strain that is occurring naturally in Brazil.

## Methods

### Collection and morphological identification

The insects were collected at two sites. The first collection was of individuals parasitizing *S. frugiperda* eggs on corn in the experimental area on the “Luiz de Queiroz” College of Agriculture campus in Piracicaba, São Paulo State, in 2019 (22° 41′ 53″ S, 47° 38′ 30″ W). In the second site, *S. cosmioides* eggs were collected from Bt transgenic soybeans, cultivar M5917IPRO, during the 2018–2019 harvest at São José dos Pinhais, Paraná State, in southern Brazil (25° 36′ 25.2″ S, 49° 08′ 04.2″ W). These sites are 358 km apart. Parasitized eggs were placed in glass tubes until adult parasitoids emerged. Twenty-four hours after emergence, the adult specimens were killed and preserved in absolute ethanol for morphological and molecular identification.

Identification was based mainly on the male genitalia, prepared and mounted on microscope slides according to the method of Polaszek and Kimani^21^. The *T. remus* individuals collected in Brazil were compared morphologically and molecularly with specimens of laboratory rearing colony from Venezuela (source material from Trinidad and Tobago) that have been reared in the Laboratory of Insect Biology of USP/ESALQ since 2011. Individuals from each field collection and from the laboratory rearing colony were deposited in the Oscar Monte Entomophagous Insect Collection at the Biological Institute in Campinas, São Paulo, Brazil, under the following numbers: IBCBE 003669, IBCBE 003671, IBCBE 003672, IBCBE 003674, IBCBE 003677–IBCBE 003687; and in the collection of the ESALQ Entomology Museum (MELQ), numbers ESALQENT000553–ESALQENT000562 (Table 1).

**Table 1:** Voucher specimens deposited in the Oscar Monte Entomophagous Insect Collection at the Instituto Biológico in Campinas, São Paulo, Brazil (IBCBE 003669, IBCBE and in the collection of the ESALQ Entomology Museum (MELQ).

Code no	Sex	City	State	Latitude	Longitude	Host plant	Host	Mounting methods
IBCBE 003668	Male	Jaguariúna*	São Paulo	22° 42′ 21″ S	46° 59′ 08″ W	*Zea mays*	*Spodoptera frugiperda*	Slide**
IBCBE 003669	Male	Jaguariúna*	São Paulo	22° 42′ 21″ S	46° 59′ 08″ W	*Zea mays*	*Spodoptera frugiperda*	Slide
IBCBE 003670	Female	Jaguariúna*	São Paulo	22° 42′ 21″ S	46° 59′ 08″ W	*Zea mays*	*Spodoptera frugiperda*	Slide**
IBCBE 003671	Female	Jaguariúna*	São Paulo	22° 42′ 21″ S	46° 59′ 08″ W	*Zea mays*	*Spodoptera frugiperda*	Point
IBCBE 003672	Female	Jaguariúna*	São Paulo	22° 42′ 21″ S	46° 59′ 08″ W	*Zea mays*	*Spodoptera frugiperda*	Point
IBCBE 003673	Female	Jaguariúna*	São Paulo	22° 42′ 21″ S	46° 59′ 08″ W	*Zea mays*	*Spodoptera frugiperda*	Slide**
IBCBE 003674	Male	Piracicaba	São Paulo	22° 41′ 53″ S	47° 38′ 30″ W	*Zea mays*	*Spodoptera frugiperda*	Slide**
IBCBE 003675	Male	Piracicaba	São Paulo	22° 41′ 53″ S	47° 38′ 30″ W	*Zea mays*	*Spodoptera frugiperda*	Slide
IBCBE 003676	Female	Piracicaba	São Paulo	22° 41′ 53″ S	47° 38′ 30″ W	*Zea mays*	*Spodoptera frugiperda*	Slide**
IBCBE 003677	Female	Piracicaba	São Paulo	22° 41′ 53″ S	47° 38′ 30″ W	*Zea mays*	*Spodoptera frugiperda*	Slide**
IBCBE 003678	Male	Piracicaba	São Paulo	22° 41′ 53″ S	47° 38′ 30″ W	*Zea mays*	*Spodoptera frugiperda*	Point
IBCBE 003679	Male	Piracicaba	São Paulo	22° 41′ 53″ S	47° 38′ 30″ W	*Zea mays*	*Spodoptera frugiperda*	Point
IBCBE 003680	Male	São José dos Pinhais	Paraná	25°36′25.2″ S	49° 08′ 04.2″ W	*Glycine max*	*Spodoptera cosmioides*	Slide
IBCBE 003681	Male	São José dos Pinhais	Paraná	25°36′25.2″ S	49° 08′ 04.2″ W	*Glycine max*	*Spodoptera cosmioides*	Slide
IBCBE 003682	Female	São José dos Pinhais	Paraná	25°36′25.2″ S	49° 08′ 04.2″ W	*Glycine max*	*Spodoptera cosmioides*	Slide
IBCBE 003683	Female	São José dos Pinhais	Paraná	25°36′25.2″ S	49° 08′ 04.2″ W	*Glycine max*	*Spodoptera cosmioides*	Slide
IBCBE 003684	Male	São José dos Pinhais	Paraná	25°36′25.2″ S	49° 08′ 04.2″ W	*Glycine max*	*Spodoptera cosmioides*	Point
IBCBE 003685	Male	São José dos Pinhais	Paraná	25°36′25.2″ S	49° 08′ 04.2″ W	*Glycine max*	*Spodoptera cosmioides*	Point
IBCBE 003686	Female	São José dos Pinhais	Paraná	25°36′25.2″ S	49° 08′ 04.2″ W	*Glycine max*	*Spodoptera cosmioides*	Point
IBCBE 003687	Female	São José dos Pinhais	Paraná	25°36′25.2″ S	49° 08′ 04.2″ W	*Glycine max*	*Spodoptera cosmioides*	Point
ESALQENT000553	Female	São José dos Pinhais	Paraná	25°36′25.2″ S	49° 08′ 04.2″ W	*Glycine max*	*Spodoptera cosmioides*	Slide
ESALQENT000554	Female	São José dos Pinhais	Paraná	25°36′25.2″ S	49° 08′ 04.2″ W	*Glycine max*	*Spodoptera cosmioides*	Slide
ESALQENT000555	Male	São José dos Pinhais	Paraná	25°36′25.2″ S	49° 08′ 04.2″ W	*Glycine max*	*Spodoptera cosmioides*	Point
ESALQENT000556	Female	São José dos Pinhais	Paraná	25°36′25.2″ S	49° 08′ 04.2″ W	*Glycine max*	*Spodoptera cosmioides*	Point
ESALQENT000557	Female	Jaguariúna	São Paulo	22° 42′ 21″ S	46° 59′ 08″ W	*Zea mays*	*Spodoptera frugiperda*	Slide
ESALQENT000558	Female	Jaguariúna	São Paulo	22° 42′ 21″ S	46° 59′ 08″ W	*Zea mays*	*Spodoptera frugiperda*	Point
ESALQENT000559	Male	Jaguariúna	São Paulo	22° 42′ 21″ S	46° 59′ 08″ W	*Zea mays*	*Spodoptera frugiperda*	Point
ESALQENT000560	Female	Piracicaba	São Paulo	22° 41′ 53″ S	47° 38′ 30″ W	*Zea mays*	*Spodoptera frugiperda*	Slide
ESALQENT000561	Female	Piracicaba	São Paulo	22° 41′ 53″ S	47° 38′ 30″ W	*Zea mays*	*Spodoptera frugiperda*	Slide
ESALQENT000562	Male	Piracicaba	São Paulo	22° 41′ 53″ S	47° 38′ 30″ W	*Zea mays*	*Spodoptera frugiperda*	Point
ESALQENT000563	Male	São José dos Pinhais	Paraná	25°36′25.2″ S	49° 08′ 04.2″ W	*Glycine max*	*Spodoptera cosmioides*	Slide**
ESALQENT000564	Male	São José dos Pinhais	Paraná	25°36′25.2″ S	49° 08′ 04.2″ W	*Glycine max*	*Spodoptera cosmioides*	Slide**
ESALQENT000565	Male	Jaguariúna	São Paulo	22° 42′ 21″ S	46° 59′ 08″ W	*Zea mays*	*Spodoptera frugiperda*	Slide**
ESALQENT000566	Male	Jaguariúna	São Paulo	22° 42′ 21″ S	46° 59′ 08″ W	*Zea mays*	*Spodoptera frugiperda*	Slide**
ESALQENT000567	Male	Piracicaba	São Paulo	22° 41′ 53″ S	47° 38′ 30″ W	*Zea mays*	*Spodoptera frugiperda*	Slide**
ESALQENT000568	Male	Piracicaba	São Paulo	22° 41′ 53″ S	47° 38′ 30″ W	*Glycine max*	*Spodoptera frugiperda*	Slide**

*Telenomus remus introduced from Venezuela by Embrapa Jaguariúna (source material Trinidad and Tobago).

**Telenomus remus used for molecular extraction and then mounted on microscope slide.

For double mounting of individuals obtained through non-destructive extraction, the specimens must be dehydrated in a critical-point dryer^22^ to prevent the antennae, head and gaster from collapsing. Male genitalia were removed, mounted in Canada balsam^21,23^, and appropriately labeled. Photographs were taken with a Leica M165C stereomicroscope equipped with a Leica DFC 420 digital camera and a dome for light scattering^24^. Image stacks were combined using Leica Application Suite v3.8 to obtain the final images with extended focus.

### DNA extraction

To obtain the DNA barcodes we used six *T. remus* specimens: a female and male from Piracicaba, a male from São José dos Pinhais, and two females and a male from the colony at Laboratory of Insect Biology of USP/ESALQ (originally from Venezuela rearing colony). Total genomic DNA *T. remus* from Piracicaba and Venezuela population were extracted using a non-destructive method adapted from Gilbert et al.^25^ successfully optimized to extract small arthropods such as mites, thrips, and micro-hymenopterans. The exception was the DNA extraction from São José dos Pinhais insects, which was carried out by the destructive protocol of Kenis et al.^16^ by the company GoGenetic, PR, Brazil.

The non-destructive extraction method consisted of the following steps: a single parasitoid was placed in a 1.5-mL microtube and immersed in 200 µL of digestion buffer [3 mM CaCl_2_, 2% sodium dodecyl sulfate (SDS), 40 mM dithiothreitol (DTT), 100 mM Tris pH 8 buffer, and 100 mM NaCl]. 10 µL of proteinase K (20 mg/mL) was added directly to the microtube and incubated for 20–24 h at 65 °C in the water bath. Twelve hours after the start of the incubation, 5 µL of proteinase K (20 mg/mL) was added. After the incubation period, the parasitoid was transferred to a new tube containing 80% ethanol. After 30 min, the 80% ethanol was removed and 99.5% ethanol was added. The specimen was stored at − 20 °C for subsequent mounting and morphological identification.

To continue the extraction, in the solution resulting from incubating the insect in the extraction buffer, 500 µL of CIA (chloroform + isoamyl alcohol (24:1) was added, mixed gently by inversion, and centrifuged at 13,000 RPM for 20 min. The supernatant was transferred to a new microtube and 1/10 of the total volume of sodium acetate (3 M, pH 5.2) and 3 μL of glycogen (5 mg/mL) were added. 0.7X of cold 100% isopropanol was added to the volume of DNA (the volume of isopropanol was calculated after addition of sodium acetate and glycogen). This solution was mixed gently by inversion and incubated at − 20 °C for 24 to 48 h. It was centrifuged at 14,000 rpm/35 min at 4 °C and the liquid phase was discarded. 500 μL of cold 70% ethanol was added and the solution was centrifuged at 14,000 rpm/10 min at 4 °C. The liquid phase was discarded and 500 µL of cold 95% ethanol was added. This was centrifuged at 14,000 rpm/15 min at 4 °C. The liquid phase was again discarded and the pellet was air-dried in a flow chamber for approximately 1–2 h. 40 µL of MilliQ H_2_O was added to elute and suspend the DNA. The sample was stored at − 20 °C.

### Amplification and sequencing of the COI gene fragment

The mitochondrial cytochrome c oxidase subunit I (COI) fragment, corresponding to the barcode region^26^, was amplified by polymerase chain reaction (PCR) using the primers LCO1490 and HCO2198^27^ and/or specific primers designed here, Telen-F1 (AGGATCAGCAATAAGAGCATT) and Telen-R1 (TACTGGATCTCCTCCTCCTG). The primers were designed in the Primer3 program using five *Telenomus* COI sequences from our database and two additional sequences from the Barcode of Life Data System (ADS8099; ADM4100).

The polymerase chain reactions (PCR) were carried out in a final volume of 25 µL, following the method described by Gariepy et al.^28^. The minimum amount of genomic DNA added to reactions to obtain PCR amplicons was 40 ng. The amplification conditions were 94 °C for 3 min for primary denaturation, then 35 cycles at 94 °C for 45 s, 57 °C for 45 s, and 72 °C for 1.5 min, with a final extension at 72 °C for 15 min. The amplicons were observed under ultraviolet light, after electrophoresis on 1.5% agarose gel stained with SYBR Safe (Life Technologies). The PCR purification was performed using 1 µL (20 U µL^–1^) of Exonuclease I (Thermo Fisher Scientific™) and 2 µL (1 U µL^–1^) of thermosensitive alkaline phosphatase FastAP™ (Thermo Fisher Scientific) per10 µL of the final PCR product. The purification conditions were 37 °C for 30 min, followed by 80 °C for 15 min. The bidirectional Sanger sequencing was performed at the Animal Biotechnology of ESALQ.

### Analysis of the barcode region

The sequence chromatograms of each individual were checked, edited and aligned to produce the consensus sequence in the software Sequencher 4.8 (Gene Codes Corp., Ann Arbor, MI). The presence of NUMTs (nuclear parallels of mitochondrial origin)^29^ was observed in MEGA X^30^, following the steps described by Corrêa et al.^31^. To confirm the molecular identification of the insects collected in the field, we calculated the genetic distance among insects using the Kimura 2-parameters model (K2P) in MEGA X^30^. We submitted the sequences to BOLDSystems (www.boldsystems.org/) and NCBI/BLASTn (www.ncbi.nlm.nih.gov), and the voucher specimens used for the molecular analyses were deposited in the Oscar Monte Entomophagous Insect Collection in the Instituto Biológico, Campinas, São Paulo, Brazil. Strains of Jaguariúna: MW834424 (IBCBE 003668), MW834423 (IBCBE 003670), MW834422 (IBCBE 003673), Piracicaba: MW834425 (IBCBE 003675), MW834426 (IBCBE 003676) (Table 1). Sequence of *T. remus* collected in São José dos Pinhais was deposited under access number MW834427.

For the phylogeographic analysis, we included in our database 40 sequences from 11 countries: Ecuador (KM485691), Honduras (KM485692), USA (KM485690), Kenya (MK533757, MT465126, MT465127), Ivory Coast (MK533758), South Africa (MH681660, MH681661, MH681662, MH681663, MK533746, MK533747, MK533748, MK533749), Pakistan (KY835081), Benin (MK533750, MK533751, MK533756, MN900731, MN900732), Niger (MK533752, MK533753, MK533754), China (MN123239, MN123240, MN123241, MN123242, MN123243, MN123244), and India (KP994550, KT305960, MN814077, MN879314, MN879315, MN879316, MN913332, MW052708, MW052800, MW243584). After alignment, the sequences were trimmed to 420 bp to eliminate the missing data. The number of haplotypes was calculated in DNAsp^32^ and a haplotype network was constructed using PopART software^33^.

## Results

### Morphological identification

**Figure 1:**
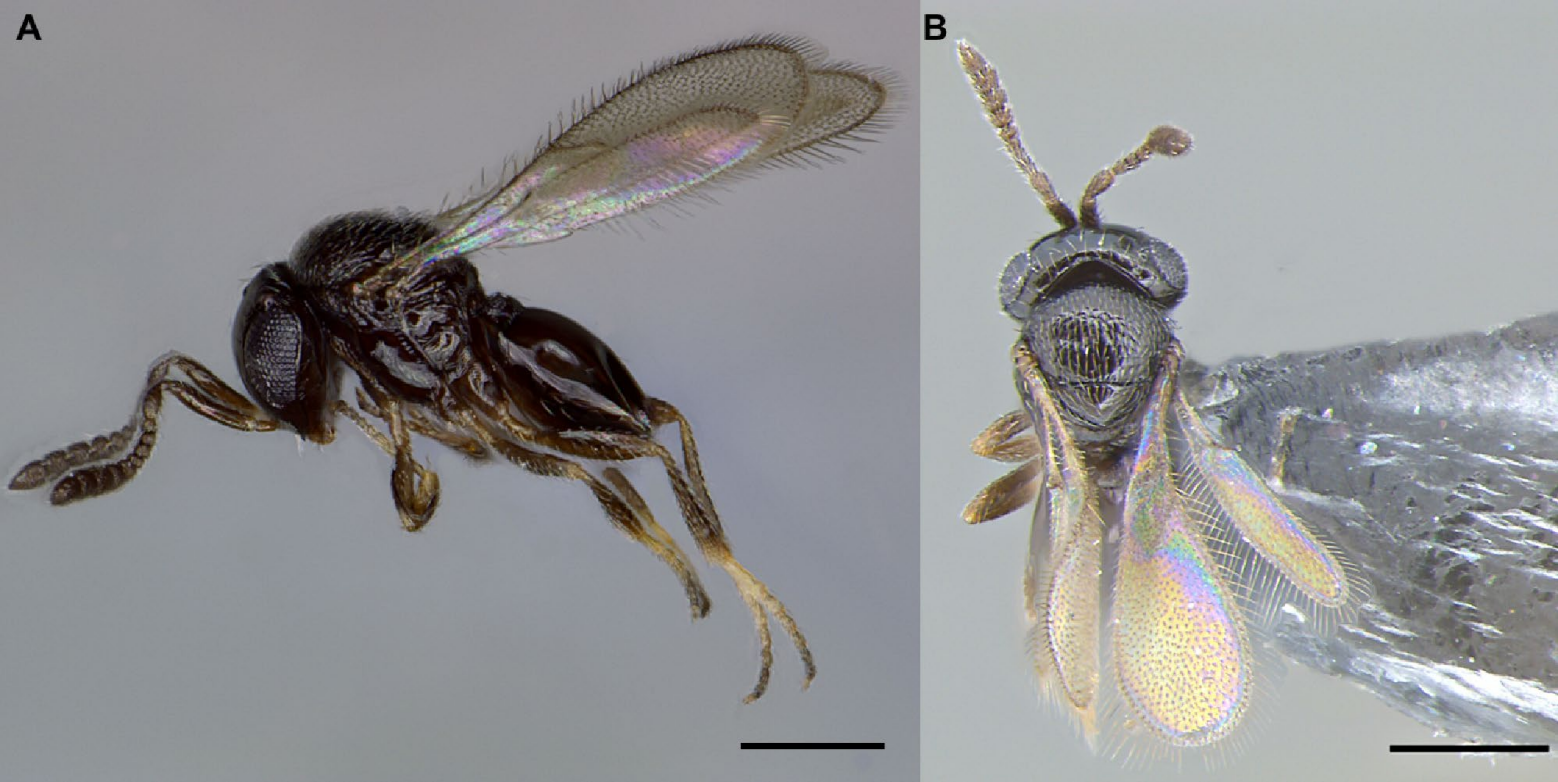
*Telenomus remus* female (scale bare: 0.20 mm): (**A**) Side view. (**B**) Dorsal view.

**Figure 2:**
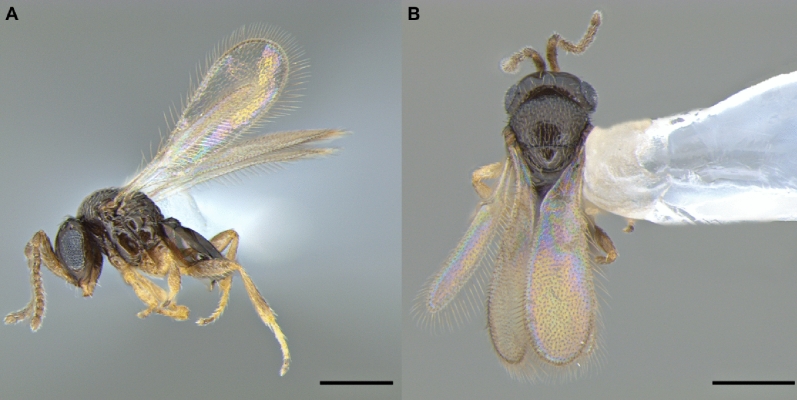
*Telenomus remus* male (scale bare: 0.20 mm): (**A**) Side view. (**B**) Dorsal view.

**Figure 3:**
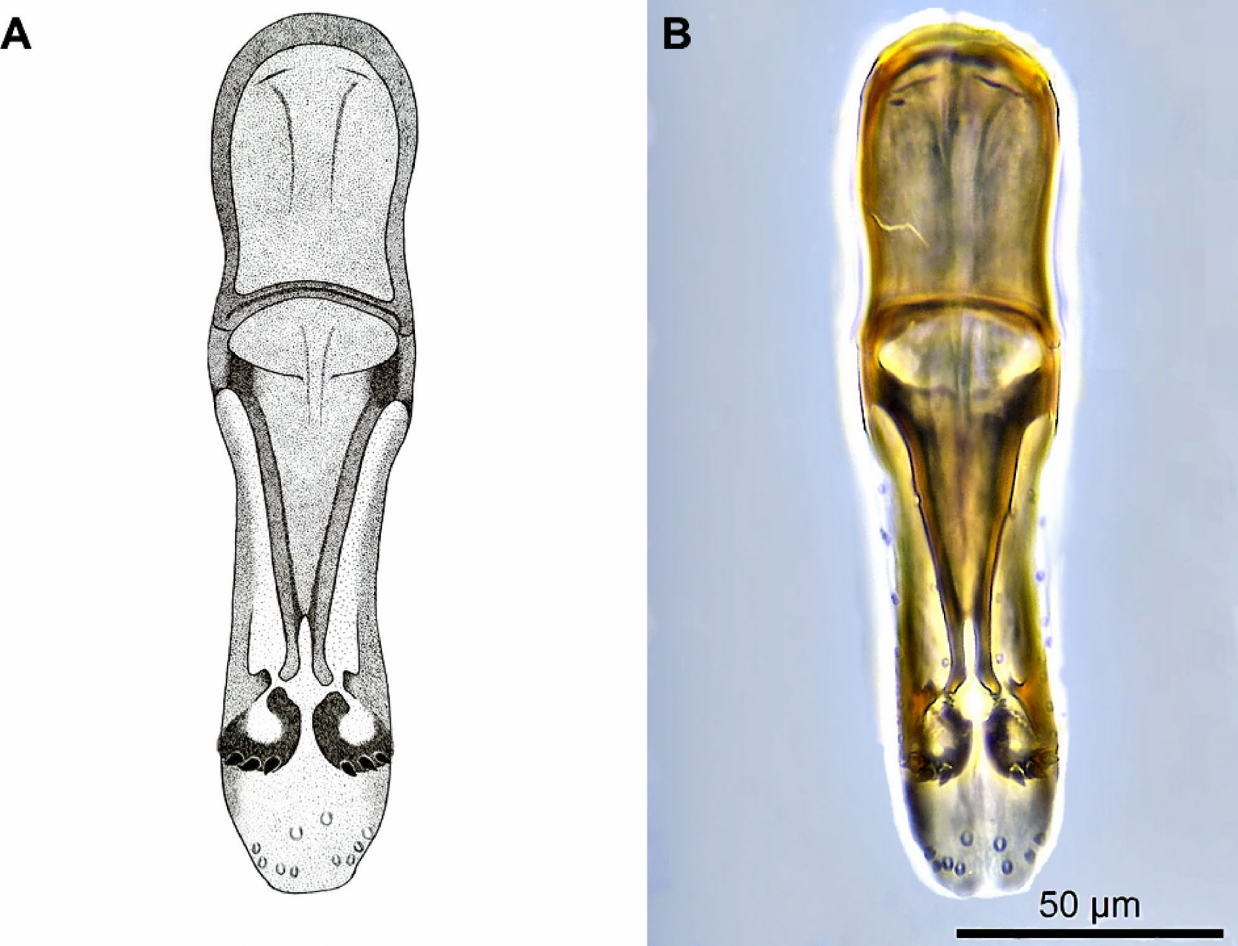
Genitalia of the male of *Telenomus remus*. (**A**) Schematic illustration. (**B**) Canada balsam preparation.

The parasitoid species was identified as *T. remus* Nixon, 1937 (Figs. 1, 2, 3) and confirmed based on the descriptions of Nixon^34^ and Chou^35^. We observed no morphological variations among individuals from Piracicaba, São José dos Pinhais, and the Venezuela laboratory strain (source material from Trinidad and Tobago).

The male has a moniliform antenna, and the legs are yellowish to brownish, lighter than in the female, which has a clavate antenna and dark brown legs (Figs. 1, 2). Genitalia of the male (Fig. 3) are rather short and broad, with three large digital teeth. Volsellar laminae are strongly pigmented. Aedeagus-volsellar shaft appears to have two rods converging toward the digiti before diverging for a short distance. On the aedeagus-volsellar shaft, in the latero-dorsal portion of digiti, is a small projection that converges with the apex of the volsellar laminae, which have no central projection. The genitalia are indistinguishable from those of *T. nawai* Ashmead, 1904 and *T. soudanensis* (Risbec, 1950)^16^.

### DNA barcoding and phylogeographic inferences

We produced a COI fragment with 527 bp from wasps collected in the field and laboratory. Six individuals had an identical barcoding region and one individual from the Venezuela laboratory strain showed a difference of one nucleotide, with an estimated genetic distance D = 0.002. Alignment of the sequences with the Barcode of Life Data Systemand BLASTn indicated > 99% similarity with other *T. remus* sequences, confirming the species identification.

**Figure 4:**
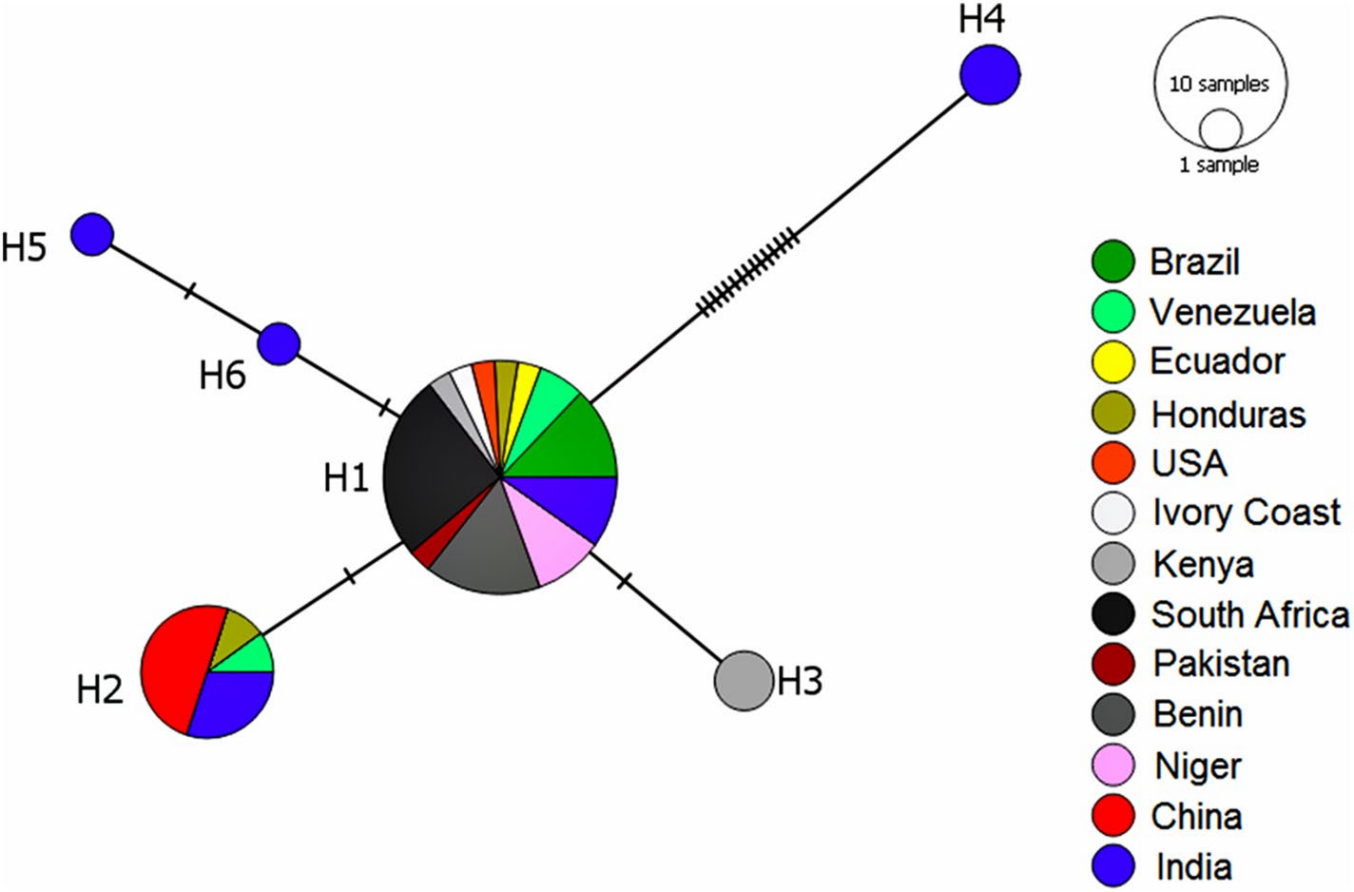
Haplotype network based on COI gene fragment sequences for *Telenomus remus* from 13 countries.

Analysis of 46 T*. remus* sequences, aligned and edited to 420 bp, generated 6 haplotypes, with the H1 haplotype present in 11 of the 13 countries with sequences available in the database, except for Honduras and China. Haplotype H2 is present in individuals from Venezuela, Honduras, China, and India. Haplotype H3 occurs in Kenya and haplotypes H4, H5, H6 occur in individuals from India (Fig. 4). The genetic relationship among the haplotypes indicates the presence of two lineages with a genetic distance of D = 0.039 among the haplotypes.

### Occurrence record

This is the first record in Brazil of natural parasitism of *T. remus* in eggs of *S. frugiperda* collected in corn and in *S. cosmioides* eggs collected in soybean, in Piracicaba, São Paulo, and São José dos Pinhais, Paraná, respectively.

## Discussion

The natural occurrence of *T. remus* parasitizing eggs of *S. frugiperda* and *S. cosmioides* opens new prospects for the use of this parasitoid in biological control programs for *Spodoptera* species in Brazil. The natural occurrence of *T. remus* is a strong indication that this parasitoid has the capacity to survive in the field and to colonize and parasitize *Spodoptera* species.

Successful establishment in a new region depends on the ability of a species to respond to the local environmental conditions and ecological interactions to which it is subjected^36,37^^.^^38^. Some species show a lag period, which corresponds to a time period to allow for the evolution/rearrangements of adaptive genes and establishment in a new region^39,40^. That may have occurred with *T. remus* in Brazil, to where this wasp species was introduced three times, in 1983, 1996, and 2011.

A similar delay occurred in Florida, USA, in the 1970s and 1980s, when *T*. *remus* was released and did not at first appear to succeed in establishing itself^41^. In 2009 and 2013, *T*. *remus* was collected in north-central Florida, approximately 500 km distant from the sites where it had been initially released, suggesting that *T. remus* individuals might have dispersed and established in that area or were accidentally introduced independently. Recently, natural occurrences of *T*. *remus* were reported in Benin, Ivory Coast, Kenya, Niger, and South Africa^16^. The exact geographic origin and introduction date of *T. remus* in Africa are not known, although the introduction occurred prior to the invasion of *S. frugiperda* because individuals of *T. remus* were collected in Kenya in 1988^16^.

The genus *Telenomus* is formed by many species complexes that can be difficult to identify, which impedes accurate reporting of natural parasitism by the correct *Telenomus* species. In particular, *T. remus,* described by Nixon^34^, has a long history of taxonomic confusion. *Telenomus remus* may be a junior synonym of *T. spodopterae* Dodd, 1913, a species collected from eggs of *Spodoptera* sp. in Indonesia^16^. However, Nixon did not use the name given by Dodd because the description was superficial, and based only on the female. *Telenomus nawai* Ashmead, 1904, *T. solitus* Johnson, 1983, and *T*. *minutissimus* Ashmead, 1895 and *T. soudanensis* Risbec have also been suggested as synonyms of *T*. *remus*, although these assumptions were not confirmed^15,16,42–44^. Many *Telenomus* species have very similar external morphology, including the male genitalia, requiring taxonomic revisions to define the morphological patterns for accurate species identification.

An integrative approach using molecular tools can be helpful for correct identification of species in the *californicus* group. However, DNA barcoding for microhymenopterans can be challenging, due to the difficulty of obtaining genomic DNA in sufficient quantity and quality from non-destructive DNA extractions of small insects. The low efficiency of universal primers for this group of organisms may also be a problem. Here, we performed a non-destructive DNA extraction capable of obtaining enough DNA to amplify a COI gene fragment and maintain the integrity of voucher insects. This methodology is recommended for DNA barcoding production since these insects could be helpful to future systematic studies and morphological identification conferences. We also designed efficient specific primers to produce amplicons and sequencing of the genus *Telenomus*, even for individuals conserved in 70% ethanol, a less than ideal condition for preserving the DNA.

DNA barcoding was extremely useful in confirming the identification of *T*. *remus*. Wasps collected in the field in Brazil had a low genetic distance from the Venezuela laboratory population (D < 0.002) and from other DNA barcoding sequences of *T. remus* obtained from online databases, confirming the identification. This integrative approach is successfully used to identify various groups of insects that are difficult to identify morphologically^45,46^ and is a recommended strategy for accurate identification of biological control agents and their associations with their hosts^28^.

The COI sequence analysis of *T. remus* revealed the presence of six haplotypes in two mitochondrial strains in America, Africa, and Asia. Haplotype H4, present in India, represents one of the *T. remus* strains, while the other five haplotypes, particularly haplotype H1, represent the other strain, which occurs worldwide. Two important hypotheses apply to the *T. remus* genetic strains. The first is that different *T. remus* genetic strains are present in the geographic center of origin of the species. This hypothesis is based on the phylogeographic concept of greater haplotype diversity where a species originates^47^. The second hypothesis is that a cryptic species close to *T. remus* is represented by haplotype H4; however, more thorough DNA barcoding analyses of the genus *Telenomus* are needed to determine if a strain of *T. remus* or another similar species occurs in India.

*Telenomus remus* was introduced into different parts of the world to aid in classic biological control of noctuid pests in the 1970s and 1980s. Specimens of this wasp were introduced into several countries by the Commonwealth Institute of Biological Control (CIBC) and the Caribbean Agricultural Research and Development Institute (CARDI). According to Cock^14^, *T. remus* populations from Papua New Guinea and the Dominican Republic were distributed in India, Pakistan, Barbados, several Caribbean Islands (Antigua, Dominica, Montserrat, St. Kitts, St. Vincent), Trinidad and Tobago, and Venezuela. We can therefore infer that the parasitoids collected in Brazil originated from this strain introduced in the Americas.

The recapture of *T. remus* in the field opens prospects for new bioecological and parasitism studies on *Spodoptera* species and other noctuids in field conditions. However, up to the present, the *T. remus* strains maintained in the laboratory have not been successful in colonization and have shown a low rate of parasitism in biological control programs for crops in Brazil^48–51^^.^

*Spodoptera frugiperda* has achieved the status of a cosmopolitan megapest. Prospecting for biological-control agents that would efficiently control this pest would have a global impact, particularly in view of its history of resistance to insecticides and Bt plants. We suggest that the *T. remus* strain distributed by anthropogenic action into the Americas, Africa, and Asia is able to adapt to different agricultural landscapes. Therefore, reintroductions of natural populations of *T. remus* from different geographical origins may be an efficient tactic for classical and augmentative biological control of *S. frugiperda* in different parts of the world. Accurate identification of *T. remus* and its genetic strains is essential for the success of biological control programs using this parasitoid.

## Data Availability

All data generated or analysed during this study are included in this published article.

## References

[1] Cruz, I. *A lagarta-do-cartucho na cultura do milho* (EMBRAPA, CNPMS, 1995).

[2] Nagoshi RN (2009). Can the amount of corn acreage predict fall armyworm (Lepidoptera: Noctuidae) infestation levels in nearby cotton?. J. Econ. Entomol..

[3] Bernardi O (2014). Low susceptibility of *Spodoptera cosmioides*, *Spodoptera eridania* and *Spodoptera frugiperda* (Lepidoptera: Noctuidae) to genetically-modified soybean expressing Cry1Ac protein. Crop Prot..

[4] Machado EP (2020). Cross-crop resistance of *Spodoptera frugiperda* selected on *Bt* maize to genetically-modified soybean expressing Cry1Ac and Cry1F proteins in Brazil. Sci. Rep..

[5] Sparks AN (1979). A review of the biology of the fall armyworm. Fla. Entomol..

[6] Knipling EF (1980). Regional management of the fall armyworm – a realistic approach?. Fla. Entomol..

[7] Georgen G, Lava Kumar P, Sankung SB, Togola A, Tamó M (2016). First report of outbreaks of the fall armyworm *Spodoptera frugiperda* (J E Smith) (Lepidoptera, Noctuidae), a new alien invasive pest in West and Central Africa. PLoS ONE.

[8] Caniço A, Mexia A, Santos L (2020). Seasonal dynamics of the alien invasive insect pest *Spodoptera frugiperda* Smith (Lepidoptera: Noctuidae) in Manica Province, central Mozambique. Insects.

[9] Botha AS, Erasmus A, du Plessis H, Van den Berg J (2019). Efficacy of Bt maize for control of *Spodoptera frugiperda* (Lepidoptera: Noctuidae) in South Africa. J. Econ. Entomol..

[10] Lira EC (2020). Resistance of *Spodoptera frugiperda* (Lepidoptera: Noctuidae) to spinetoram: inheritance an cross-resistance to Spinosad. Pest Manag. Sci..

[11] Boaventura D (2020). Detection of a ryanodine receptor target-site mutation in diamide insecticide resistant fall armyworm Spodoptera frugiperda. Pest Manag. Sci..

[12] Carvalho RA, Omoto C, Field LM, Williamson MS, Bass C (2013). Investigating the molecular mechanisms of organophosphate and pyrethroid resistance in the fall armyworm *Spodoptera frugiperda*. PLoS ONE.

[13] Omoto C (2016). Field-evolved resistance to Cry1Ab maize by *Spodoptera frugiperda* in Brazil. Pest Manag. Sci..

[14] Cock, M. J. W. *A Review of Biological Control of Pests in the Commonwealth Caribbean and Bermuda up to 1982* (Commonwealth Institute of Biological Control Technical Communication, Farnham Royal United Kingdom, 1985).

[15] Cave RD (2000). Biology, ecology and use in pest management of *Telenomus remus*. Biocontrol News and Information.

[16] Kenis M (2019). *Telenomus remus*, a candidate parasitoid for the biological control of *Spodoptera frugiperda* in Africa, is already present on the continent. Insects.

[17] Danks HV (1988). Systematics in support of entomology. Annu. Rev. Entomol..

[18] Zucchi RA, Parra JRP, Botelho OSM, Côrrea-Ferreira BS, Bento JM (2002). Taxonomia e controle biológico de pragas. Controle Biológico no Brasil: Parasitoides e Predadores.

[19] Hernández D, Ferrer F, Linares B (1989). Introducción de Telenomus remus Nixon (Hymenoptera.: Scelionidae) para controlar Spodoptera frugiperda (Lep.: Noctuidae) en Yaritagua Venezuela. Agron. Trop..

[20] Naranjo-Guevara N, Santos LAOD, Barbosa NCCP, Corrêa e Castro ACM, Fernandes OA (2020). Long-term mass rearing impacts performance of the egg parasitoid *Telenomus remus* (Hymenoptera: Platygastridae). J. Entomol. Sci..

[21] Polaszek A, Kimani SW (1990). *Telenomus* species (Hymenoptera: Scelionidae) attacking eggs of pyralid pests (Lepidoptera) in Africa: a review and guide to identification. Bull. Entomol. Res..

[22] Gordh G, Hall JC (1979). A critical point drier used as a method of mounting insects from alcohol. Entomol. News.

[23] Johnson NF (1984). Systematics of Nearctic Telenomus: classification and revisions of the Podisi and Phymatae species groups (Hymenoptera, Scelionidae).

[24] Kerr PH, Fisher EM, Buffington ML (2008). Dome lighting for insect imaging under a microscope. Am. Entomol..

[25] Gilbert MTP, Moore W, Melchior L, Worobey M (2007). DNA extraction from dry museum beetles without conferring external morphological damage. PLoS ONE.

[26] Hebert PDN, Ratnasingham S, deWaard JR (2003). Barcoding animal life: cytochrome *c* oxidase subunit 1 divergences among closely related species. Proc. R. Soc. B Biol. Sci..

[27] Folmer O, Black M, Hoeh W, Lutz R, Vrijenhoek R (1994). DNA primers for amplification of mitochondrial cytochrome c oxidase subunit I from diverse metazoan invertebrates. Mol. Mar. Biol. Biotech..

[28] Gariepy TD, Haye T, Zhang J (2014). A molecular diagnostic tool for the preliminary assessment of the host-parasitoid associations in biological control programmes for a new invasive pest. Mol. Ecol..

[29] Lopez JV, Yuhki N, Masuda R, Modi W, O’Brien SJ (1994). *Numt,* a recent transfer and tandem amplification of mitochondrial DNA to the nuclear genome of the domestic cat. J. Mol. Evol..

[30] Kumar S, Stecher G, Li M, Knyaz C, Tamura K (2018). Mega X: molecular evolutionary genetics analysis across computing platforms. Mol. Biol. Evol..

[31] Corrêa AS, Vinson CC, Braga LS, Guedes RNC, Oliveira LO (2017). Ancient origin and recent range expansion of the maize weevil Sitophilus zeamais, and its genealogical relationship to the rice weevil *S. oryzae*. Bull. Entomol. Res.

[32] Rozas J (2017). DnaSP 6: DNA Sequence Polymorphism analysis of large datasets. Mol. Biol. Evol..

[33] Leigh JW, Bryant D (2015). PopART: Full-feature software for haplotype network construction. Methods Ecol. Evol..

[34] Nixon GEJ (1937). Some Asiatic Telenominae (Hym., Proctotrupoidea). Ann. Mag. Nat. Hist..

[35] Chou LY (1987). Note on *Telenomus remus* (Hymenoptera: Scelionidae). Bull. Soc. Entomol..

[36] Hall RW, Ehler LE (1979). Rate of establishment of natural enemies in classical biological control. Bull. Entomol. Soc. Am..

[37] Fernández-Arhex V, Corley JC (2003). The functional response of parasitoids and its implications for biological control. Biocontrol Sci. Technol..

[38] Tougeron K, Brodeur J, Le Lann C, van Baaren J (2020). How climate change affects the seasonal ecology of insect parasitoids. Ecol. Entomol..

[39] Ellstrand NC, Schierenbeck K (2000). Hybridization as a stimulus for the evolution of invasiveness in plants?. Proc. Natl. Acad. Sci. USA.

[40] Mack RN (2000). Biotic invasions: causes, epidemiology, global consequences, and control. Ecol. Appl..

[41] Hay-Roe MM, Nagoshi RN, Meagher RL, De Lopez MA, Trabanino R (2015). Isolation and DNA barcode characterization of a permanent *Telenomus* (Hymenoptera: Platygastridae) population in Florida that targets fall armyworm (Lepidoptera: Noctuidae). Ann. Entomol. Soc. Am..

[42] Braithwaite, C. W. D. & Pollard, G. V. (eds.) *Urgent Plant Pest and Disease Problems in the Caribbean* (InterAmerican Institute for Cooperation on Agriculture, Ocho Rios, Jamaica, 1981).

[43] Yaseen M, Bennett FD, Barrow RM, Braithwaite CWD, Pollard GV (1981). Introduction of exotic parasites for control of *Spodoptera frugiperda* in Trinidad, the Eastern Caribbean and Latin America. Urgent Plant Pest and Disease Problems in the Caribbean.

[44] Centre for Agriculture Bioscience International (CABI). *Plantwise Technical Factsheet: Telenomus*. http://www.plantwise.org (2014).

[45] Shimbori EM (2020). Two new species of *Nealiolus* Mason (Hymenoptera, Braconidae, Brachistinae) reared from pest weevils (Coleoptera, Curculionidae). Zootaxa.

[46] Polaszek A (2021). *Telenomus nizwaensis* (Hymenoptera: Scelionidae), an important egg parasitoid of the pomegranate butterfly *Deudorix livia* Klug (Lepidoptera: Lycaenidae) in Oman. PLoS ONE.

[47] Avise JC (1987). Intraspecific phylogeography: the mitochondrial DNA bridge between population genetics and systematics. Ann. Rev. Ecol. Syst..

[48] Bueno RCOF (2010). Parasitism capacity of *Telenomus remus* Nixon (Hymenoptera: Scelionidae) on *Spodoptera frugiperda* (Smith) (Lepidoptera: Noctuidae) eggs. Braz. Arch. Biol. Technol..

[49] Pomari AF, Bueno AF, Bueno RCOF, Menezes Junior AO (2012). Biological characteristics and thermal requirements of the biological control agent *Telenomus remus* (Hymenoptera: Platygastridae) reared on eggs of different species of the genus *Spodoptera* (Lepidoptera: Noctuidae). Ann. Entomol. Soc. Am..

[50] Bueno RCOF, Bueno AF, Xavier MFC, Carvalho MM (2014). *Telenomus remus* (Hymenoptera: Platygastridae) parasitism on eggs of *Anticarsia gemmatalis* (Lepidoptera: Erebidae) compared with its natural host *Spodoptera frugiperda* (Lepidoptera: Noctuidae). Ann. Entomol. Soc. Am..

[51] Queiroz AP (2017). Quality control of *Telenomus remus* (Hymenoptera: Platygastridae) reared on the factitious host *Corcyra cephalonica* (Lepidoptera: Pyralidae) for successive generations. Bull. Entomol. Res..

